# Mapping regional variability of exclusive breastfeeding and its determinants at different infant’s age in Tanzania

**DOI:** 10.1186/s12884-023-06076-5

**Published:** 2023-11-03

**Authors:** Ola Farid Jahanpour, Elphas Luchemo Okango, Jim Todd, Henry Mwambi, Michael J. Mahande

**Affiliations:** 1 Department of Epidemiology and Biostatistics, Institute of Public Health, Kilimanjaro Christian Medical University College (KCMUCo), Moshi, Tanzania; 2 Department of Mathematical Sciences, Strathmore University, Nairobi, Kenya; 3 Department of Population Health, London School of Hygiene and Tropical Medicine, London, UK; 4 National Institute for Medical Research, Mwanza, Tanzania; 5 School of Mathematics, Statistics & Computer Science, University of KwaZulu-Natal, Pietermaritzburg, South Africa

**Keywords:** Exclusive breastfeeding, Tanzania, Generalized linear mixed models, Demographic health survey, Secondary data analysis

## Abstract

**Introduction:**

Despite its numerous benefits, exclusive breastfeeding (EBF) remains an underutilized practice. Enhancing EBF uptake necessitates a focused approach targeting regions where its adoption is suboptimal. This study aimed to investigate regional disparities in EBF practices and identify determinants of EBF among infants aged 0–1, 2–3, and 4–5 months in Tanzania.

**Methods:**

This cross-sectional study utilized data from the 2015/16 Tanzania Demographic and Health Survey. A total of 1,015 infants aged 0–5 met the inclusion criteria, comprising 378 aged 0–1 month, 334 at 2–3 months, and 303 at 4–5 months. EBF practices were assessed using a 24-hour recall method. A generalized linear mixed model, with fixed covariates encompassing infant and maternal attributes and clusters for enumeration areas (EAs) and regions, was employed to estimate EBF proportions.

**Results:**

Regional disparities in EBF were evident among infants aged 0–1, 2–3, and 4–5 months, with decline in EBF proportions as an infant’s age increases. This pattern was observed nationwide. Regional and EA factors influenced the EBF practices at 0–1 and 2–3 months, accounting for 17–40% of the variability at the regional level and 40–63% at the EA level. Literacy level among mothers had a significant impact on EBF practices at 2–3 months (e.g., women who could read whole sentences; AOR = 3.2, 95% CI 1.1,8.8).

**Conclusion:**

Regional disparities in EBF proportions exist in Tanzania, and further studies are needed to understand their underlying causes. Targeted interventions should prioritize regions with lower EBF proportions. This study highlights the clustering of EBF practices at 0–1 and 2–3 months on both regional and EA levels. Conducting studies in smaller geographical areas may enhance our understanding of the enablers and barriers to EBF and guide interventions to promote recommended EBF practices.

**Supplementary Information:**

The online version contains supplementary material available at 10.1186/s12884-023-06076-5.

## Introduction

A newborn is universally cherished across cultures, deserving an environment that promotes holistic well-being, as defined by the World Health Organization—comprising physical, mental, and social facets, beyond mere disease absence. Exclusive breastfeeding (EBF), defined as “feeding a baby solely with breast milk, excluding all other foods or liquids, except medications or supplements”, offers profound potential in nurturing this well-being. This practice, endorsed for an infant’s initial six months [1], confers an array of physical advantages: bolstering survival rates [2], safeguarding against infections like respiratory and gastrointestinal illnesses, enhancing immunity [3], and fulfilling the nutritional requirements essential for growth and development [3]. Breast milk plays a pivotal role in the development of newborn babies, aiding crucial functions such as sucking, and swallowing. When consuming breast milk digestion, absorption, and renal functions are not harmed [3]. Moreover, breastfeeding is linked to cognitive development, resulting in improved intelligence test scores among children and adolescents [4]. Its eco-friendliness further underscores its significance; breast milk’s production, storage, and disposal are deemed more environmentally sustainable than alternative infant meals [5]. Despite these manifold benefits, global EBF proportions remain below 50% [6], with Tanzania reporting 59% exclusivity [7].

To enhance the prevalence of EBF, optimizing the use of our often limited resources becomes paramount. This optimization hinges on a strategic approach, targeting regions and areas that exhibit suboptimal EBF proportions while pinpointing the most opportune timing for interventions. Variations in EBF practices are multifaceted and have been ascribed to several factors, including regional disparities within a country [8–12], the distinction between rural and urban environments [5, 13], altitude-related disparities [10], seasonal influences [14], and contextual factors intricately linked to a particular geographic region or community’s way of life [15–18]. Notably, our analysis of EBF prevalence across Tanzania has unveiled distinctive geographical patterns [18].

Another critical set of determinants revolves around infant-related characteristics, with particular emphasis on the infant’s age. Globally, there is a consistent pattern of declining EBF proportions as an infant advances in age [9–11, 19, 20]. This trend underscores the significance of pinpointing the precise age at which EBF practices are most likely to diminish, as it can serve as a pivotal guide for targeted interventions. The 2015/16 Tanzania Demographic and Health Survey (DHS) report provides compelling evidence of this age-related variance in EBF prevalence. It reveals that EBF proportions fluctuate significantly depending on the age of the infant, with 0–1 months demonstrating a substantial 84% adherence, 2–3 months exhibiting a reduced but still notable 58.8%, and a considerable decline to 26.6% for infants aged 4–5 months [7]. This observed pattern underscores the critical importance of understanding precisely the determinants of EBF when it declines, thus enabling us to tailor interventions to these vulnerable periods.

The aims of this study are to:

1. Develop prevalence maps of EBF practices at regional level at 0–1, 2–3 and 4–5 months, accounting for the hierarchical nature of the data. And be able to identify the level of variabilities of EBF practices in Tanzania.
2. Identify the determinants of EBF practices at 0–1, 2–3 and 4–5 months in Tanzania, accounting for the hierarchical nature of data.

This study used Tanzania DHS data of 2015/16 to produce detailed information on EBF practices in Tanzania by:

1. Describing the trend of the EBF practice at regional levels. Other studies have either provided estimates at the national level which may mask regional variabilities or have provided estimates at certain regions of the country [13, 20].
2. Accounting for the multilevel nature of data when making the EBF estimates and thus reducing the potential of making type 1 error. Other studies that have used DHS data have not accounted for the hierarchical nature of the data [14, 21].

## Methods

### Study design

This study conducts a secondary data analysis utilizing the 2015/2016 Tanzania DHS dataset.

### Study settings

The United Republic of Tanzania, comprising Tanzania Mainland and Zanzibar, occupies a geographical space between longitudes 29° and 41° East and latitudes 1° and 12° South. In the year 2012, Tanzania boasted a population of 44,928,923 inhabitants, distributed across 30 regions, which represent the first administrative unit, further subdivided into 169 districts, constituting the second administrative unit. The average household size stood at 4.8 individuals per household, although this metric exhibited regional disparities, ranging from 4.8 to 3.7 across different regions. Tanzania’s climate is characterized by an annual rainfall range spanning from 750 to 1400 mm. The nation’s settlements can be broadly categorized into rural and urban areas. Rural areas predominantly revolve around agricultural activities, while urban locales are hubs of non-agricultural pursuits. The majority of Tanzania’s populace resides in rural settings, accounting for 70.9% of the total population, as opposed to the 29.1% dwelling in urban areas [22]. However, the country has witnessed a notable trend of rapid urbanization, which has seen urban population proportions rise from 13.3% to 1978 to 29.1% by 2012. This urbanization wave varies in pace across different regions, influencing settlement patterns and occasionally leading to the emergence of inadequately serviced urban areas.

Communication infrastructure in Tanzania has also undergone evolution, particularly with the proliferation of telephone services since the 1990s, providing a vital means of connectivity. The nation has a network of health facilities encompassing dispensaries, health centers, hospitals, and tertiary hospitals, with varying numbers across regions. In terms of healthcare personnel, there was an average of 1.3 health workers per 1000 population, though rural areas experienced more acute staff shortages [23]. As of 2022, Tanzania’s population has increased to approximately 61,741,120 [24], reflecting a significant demographic shift over the years. 

### Data source

The DHS is a nationally representative survey characterized by a multistage cluster sampling design. The survey’s methodology entails a systematic process, commencing with the stratification of the country based on geographical regions and the classification of urban and rural areas within each region. Subsequently, the sampling process involves the initial identification of enumeration areas (EA) and the subsequent selection of households. Throughout the analysis, sampling weights are applied to appropriately account for differences in the probability of selection at both the first and second stages of sampling.

In this survey, a total of 59 strata were delineated, encompassing 608 enumeration areas (180 urban and 428 rural). At the household level, eligibility criteria encompassed women and men of reproductive age who either resided in the household or spent the night there prior to the survey. On average, each enumeration area was associated with 86 households, from which 22 were systematically selected for inclusion in the survey.

One tool employed within the DHS framework is the woman’s questionnaire. This questionnaire encompasses inquiries pertaining to women and extends to queries regarding their children, facilitating the collection of comprehensive data encompassing fundamental demographic and health indicators, including breastfeeding practices.

### Study participants and sampling procedure

**Fig. 1:**
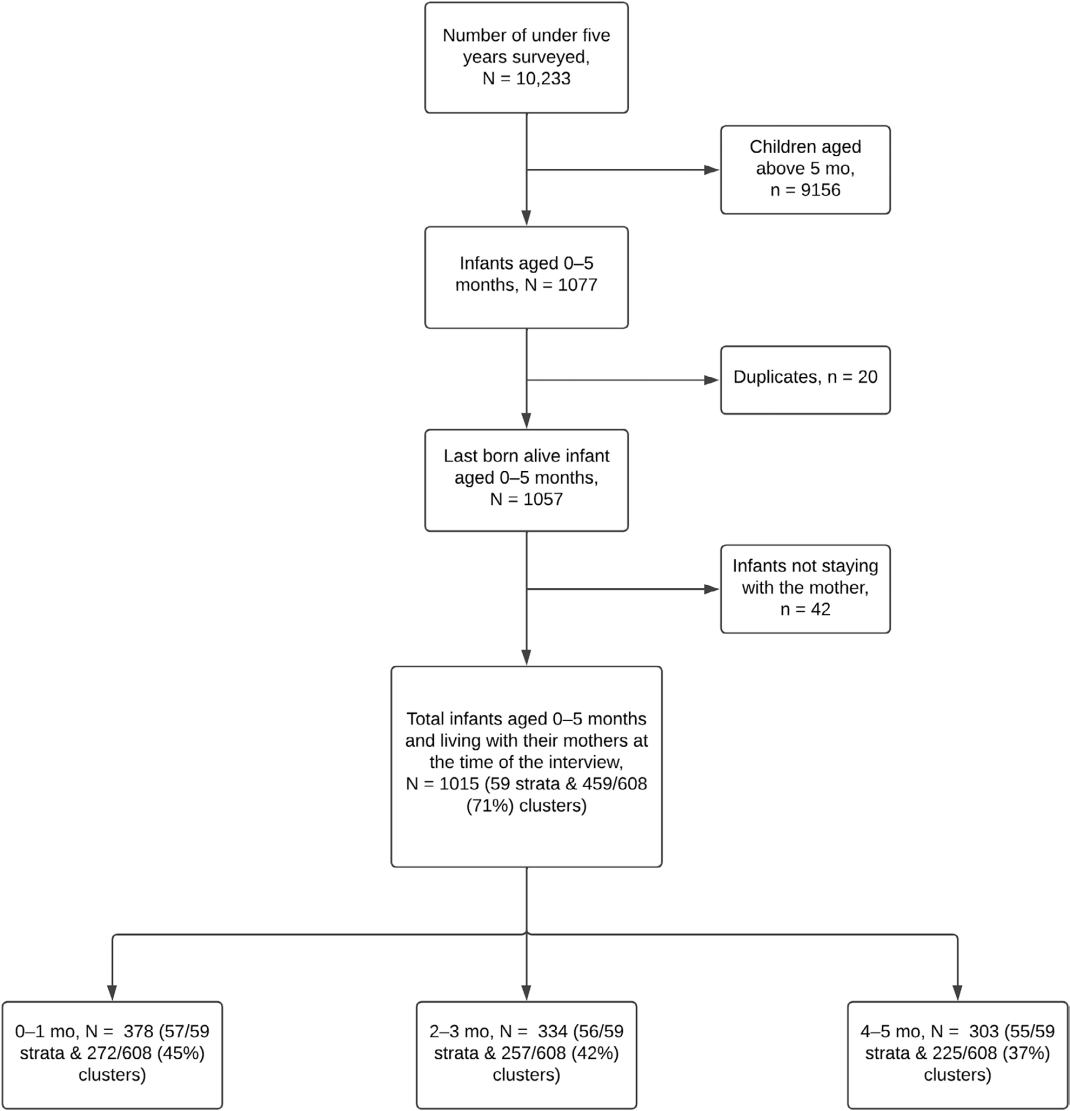
Participants’ flow

In the 2015/16 survey, a total of 13,266 women aged 15–49 were interviewed, reflecting a 97% response rate. The flow of participants is visually represented in Fig. 1. This study encompassed mothers and the youngest infant within their family who were residing with the mother at the time of the interview. The study focused on 1,015 infants falling within the age range of 0–5 months. These infants were drawn from all strata and 459 (71%) enumeration areas (EA).

For the purpose of analyzing the age group spanning from 0 to 1 month, we utilized data from 378 infants. These infants were sourced from 57 strata and 272 EA (45%). In the case of infants aged 2–3 months, the analysis involved 334 infants hailing from 56 strata and 257 EA (42%). Finally, the analysis of infants aged 4–5 months included 303 infants. These infants were drawn from 55 strata and 225 EA (37%) (Fig. 1).

### Measurements of variables

#### Response variable

The EBF was the response variable treated as a binary variable (1 “Yes”, 0 “No”). Infants exclusively breastfed in the 24 h preceding the interview were categorized as “Exclusively Breastfed” (1 “Yes”), while those who received substances other than prescribed medicines, oral rehydration solution, vitamins, and minerals were considered “Not Exclusively Breastfed” (0 “No”).

#### Exposure variables

The selection of variables and details in their categorization has already been described in another manuscript [18]. Briefly, covariate selection for the model was based on previous literature reports, data availability, and a two-stage variable selection approach (using the Chi-square test and backward selection). Infant-related variable included in the model was sex for the different infant’s age bands. Mother’s related variables included: age in years, wealth index, area of residence, literacy level, working status, whether assisted by a nurse during delivery or not, and frequency of listening to the radio and watching television (TV).

### Data processing and statistical analysis

#### Descriptive analysis

Data was analyzed using STATA version 16.0. The categorical covariates were summarized using unweighted frequencies, weighted frequencies taken from the DHS weights, proportions and 95% confidence intervals for the 0–5 months old group.

QGIS (QGIS Development Team. “QGIS Geographic Information System.” Open Source Geospatial Foundation Project. Available: https://qgis.org (accessed on 21 January 2021)) was used in map development.

#### Model selection

Three models for EBF in Tanzania were explored; a classical logistic regression model, a generalized linear mixed model (GLMM) with regions as random effects and a GLMM model with EA nested in regions. In each model, the log of odds of EBF in children was related to selected covariates, separately for children aged 0–1, 2–3 and 4–5 months of age.

Model 1 (classical logistic model): $h\left({p}_{ij}\right)={\beta }_{0}+ \varvec{\beta }\varvec{X}$

Model 2 (random effects model): $h\left({p}_{ij}\right)={\beta }_{0}+ \varvec{\beta }\varvec{X}+{\mu }_{i }$

Model 3 (nested random effects model):

$$h\left({p}_{ijk}\right)={X\beta }_{k|ij}+ X{\beta }_{ij}+{\mu }_{k|i }+ {\mu }_{i}$$

Where:

$h\left({p}_{ij}\right)$ and $h\left({p}_{ijk}\right)$ are logit link functions describing the logs of odds of EBF child $j$ in region $i$ and clusters $k$ for ages 0–1, 2–3, and 4–5

$\beta {\prime }s$ are the regression coefficients,

$X{\prime }s$ are the covariates,

${\mu }_{i}$ is the region-specific random effects,

${\mu }_{k|i }$ is the random effects capturing the variation due to different EA $k$ within a common region $i$,

${X\beta }_{k|ij}$ effect of EA k given child j is in region i,

$X{\beta }_{ij}$ regression coefficient for child j in region i.

A predicted median log of odds at the regional level was obtained from the above model. These predicted values were then used to develop maps of the prevalence of EBF of those aged 0–1, 2–3, and 4–5 for the regional level. The log of odds of EBF for each region for each of the three age bands were converted into percentages and are presented as supplementary materials (Table supplementary 1).

## Results

### Characteristics of infants aged 0–5 months and their mothers

An overview of the background characteristics of infants aged 0–5 months and their mothers is presented in Table 1. The overall proportion of EBF for infants within the 0–5 months age bracket stood at 59.2% (95% CI: 55.7–62.7%). Among the infants, there was an almost equal distribution of males and females, with EBF proportions of 51.9% and 48.1%, respectively.

**Table 1:** Background characteristics of the infants aged 0–5 months and their mothers (N = 1015)

Characteristics	Overall total (a*)		Yes EBF (b*)	
	n	%	Row %	95% CI
	1015		59.2	[55.7,62.7]
**Sex of an infant**				
Male	507	50	59	[54.2,63.7]
Female	508	50	59.4	[54.4,64.3]
**Infant’s age (month)**				
0	194	19.1	89.4	[83.7,93.3]
1	184	18.1	78.4	[71.1,84.2]
2	175	17.2	63.4	[54.9,71.2]
3	159	15.7	53.4	[44.5,62.0]
4	181	17.8	33	[25.1,41.9]
5	122	12	18.1	[11.5,27.3]
**Mother’s age (years)**				
Less than 18	49	4.8	49.6	[32.7,66.6]
18–24	411	40.5	58.4	[52.8,63.9]
25+	555	54.7	60.8	[55.7,65.7]
**Current marital status**				
Never in union/widowed/divorced/no longer living together	147	14.5	57.7	[48.4,66.5]
Married/living with partner	868	85.5	59.5	[55.7,63.2]
**Wealth index**				
Lowest	258	25.4	58.9	[51.6,65.8]
Lower	209	20.6	63	[54.5,70.8]
Middle	179	17.6	58.7	[50.5,66.4]
Higher	214	21.1	58.1	[49.3,66.3]
Highest	155	15.3	56.8	[47.2,65.9]
**Mother’s residence**				
Urban	243	23.9	51.4	[44.0,58.7]
Rural	772	76.1	62.2	[58.2,66.1]
**Literacy**				
Cannot read at all	291	28.7	53.5	[46.4,60.5]
Able to read only parts of sentence	54	5.3	49.5	[36.6,62.4]
Able to read whole sentence	670	66	62.5	[57.8,66.9]
**Who responndent works for**				
For family member/Someone else	362	35.7	62	[56.0,67.6]
Self-employed	410	40.4	61.5	[55.6,67.0]
Not working	243	23.9	51.3	[43.7,58.9]
**Assisted by nurse/midwife during delivery**				
No	420	41.4	58.3	[52.5,63.8]
Yes	595	58.6	59.8	[55.1,64.4]
**Counselled on breastfeeding:<=2 days postpartum**				
No	693	68.3	59.6	[55.4,63.6]
Yes	322	31.7	58.6	[51.5,65.3]
**Frequency of listening to radio**				
Not at all	276	27.2	60.7	[53.8,67.2]
Less than once a week	359	35.4	58.5	[52.2,64.6]
At least once a week	380	37.4	58.8	[52.6,64.7]
**Frequency of watching television**				
Not at all	597	58.8	60.1	[55.5,64.5]
Less than once a week	240	23.6	59.2	[51.7,66.3]
At least once a week	178	17.5	56	[47.4,64.2]

a* unweighted frequency, b* weighted frequency

The proportion of EBF decreases as infants age. At 0 month, 89.4% (95% CI 83.7–93.3%) of infants were exclusively breastfed, which then declined to 33% (95% CI 25.1–41.9%) at 4 months and further to 18.1% (95% CI 11.5–27.3%) at 5 months.

Regarding maternal demographics, a small percentage of mothers were under 18 years old, comprising 4.8% of the sample. Additionally, 14.5% (n = 147) of mothers were single(meaning they had never been in a union, were widowed, divorced, or were not currently living with a partner). Geographically, the majority of participants resided in rural settings, accounting for 76.1% of the sample. The proportion of EBF was higher in rural areas, standing at 62.2% (95% CI 58.2–66.1%), compared to urban areas, where it was 51.4% (95% CI 44-58.7%). Nearly a third of the mothers (28.7%) did not possess basic reading skills, while a similar percentage (23.9%) were not engaged in formal employment. Over half of the mothers (58.6%) received support from a nurse or midwife during childbirth, but only 31.7% received postpartum breastfeeding counseling.

In terms of media exposure, the majority of mothers listening to the radio (72.8%), while majority (58.8%) had never watched television.

### Prevalence of exclusive breastfeeding at different regions at 0–1, 2–3, and 4–5 months old

**Fig. 2:**
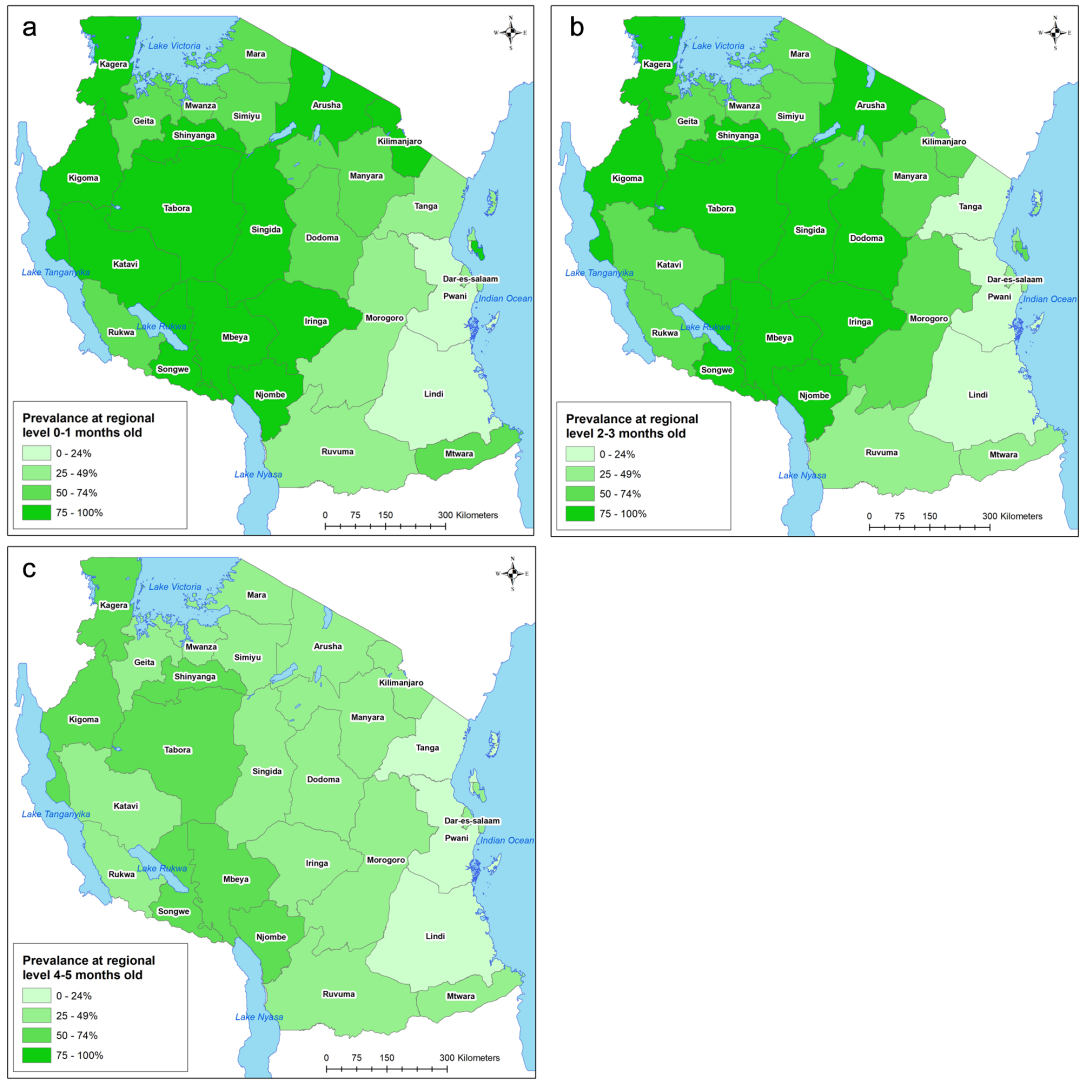
(**a**-**c**) Map of the proportion of EBF at the regional level at 0–1 month, 2–3 months and 4–5 months respectively

The variations in EBF practices across regions for infants aged 0–1, 2–3, and 4–5 months in both Tanzania mainland and Zanzibar is illustrated in Fig. 2.

For infants aged 0–1 month, the EBF proportion exhibited significant regional disparities, ranging from 94% in Mbeya to 10% in Lindi. Generally, regions along the coastal areas of the mainland displayed lower EBF proportions across all age groups. Among these coastal regions, only Mtwara exceeded a 50% EBF proportion at 0–1 months.

The data also reveals that EBF tends to decrease as infants grow older, a consistent trend observed across all regions, except for those regions that begin with a low prevalence of EBF. For instance, Mbeya had a 94% EBF proportion at both 0–1 and 2–3 months, and a decline to 64% at 4–5 months. Conversely, the Dodoma region, starting at 70% EBF at 0–1 months, managed to increase to 80% at 2–3 months.

In most cases, regions with initially low EBF prevalence displayed sporadic spikes or fluctuations in the proportion of EBF over time.

### Determinants of exclusive breastfeeding at different infant age groups

Among the three models under comparison, the model incorporating EA nested within regions exhibited superior performance, as indicated by its lower Akaike Information Criterion (AIC: 1302). In contrast, the classical logistic regression model (AIC: 1400) and the model with regions treated as random effects (AIC: 1320) displayed comparatively higher AIC values. Consequently, for all age bands considered, Model 3 was employed for further analysis.

Both the unadjusted and adjusted odds ratios derived from the final model, which incorporates random effects for both EA and regions are presented in Table 2. In the adjusted model, covariates that were adjusted for in the model were infant gender, maternal age, wealth index, area of residence, literacy level, employment status, nurse assistance during delivery, frequency of radio listenership, and frequency of television viewership, across the age groups of 0–1, 2–3, and 4–5 months. The contribution of fixed covariates to EBF is seen at 2–3 months old. At 0–1 month, EBF prevalence stands high at 84% (95% CI: 79–88%), with socio-demographic attributes exhibiting minimal impact on the practice. However, as infants progress to 2–3 months, witnessing a decline in EBF prevalence to 59% (95% CI: 53–65%), the maternal socio-demographic characteristics start to manifest their contribution to EBF practices. By the time infants reach 4–5 months, with EBF being notably scarce (Prevalence: 26%, 95% CI: 21–33%), statistical power diminishes, rendering it insufficient to discern the influence of socio-demographic attributes (Table 2).

**Table 2:** Determinants of exclusive breastfeeding among children aged 0–1, 2–3, and 4–5 months in Tanzania at a regional level from a generalized linear mixed model with EA nested in regions as random factors

Factor	0 to 1 month				2 to 3 month				4 to 5 month			
	EBF prevalence = 84% (79–88%)				EBF prevalence = 59% (52.6–64.8%)				EBF prevalence = 26% (21–33%)			
	Unadjusted Odds ratio, 95% CI	P-Value	Adjusted Odds ratio, 95% CI	P-value	Unadjusted Odds ratio, 95% CI	P-Value	Adjusted Odds ratio, 95% CI	P-value	Unadjusted Odds ratio, 95% CI	P-Value	Adjusted Odds ratio, 95% CI	P-value
**Sex of an infant**												
Male	1		1		1				1		1	
Female	0.9 (0.4,1.8)	0.688	0.9(0.4,2.2)	0.79	1.6 (0.7, 3.5)	0.241	1.5(0.7,3.4)	0.344	0.8 (0.4, 1.6)	0.512	0.8 (0.4,1.7)	0.588
**Age of the mother**												
Less than 18	1		1		1	1			1		1	
18–24	2.6 (0.5,12.7)	0.249	1.9 (0.3,12.9)	0.53	0.7 (0.1,6)	0.768	0.8 (0.1,7.4)	0.868	3.3 (0.5, 19.7)	0.196	3.1 (0.5, 20)	0.222
25+	3(0.6,14.6)	0.166	2.7(0.4,18.2)	0.313	2(0.2,16.1)	0.515	2.1 (0.2,18.8)	0.518	2.5 (0.4, 14.8)	0.309	2.5 (0.4, 15.3)	0.323
**Wealth index**												
Lowest	1		1		1	1	1		1		1	
Lower	0.5 (0.1,1.6)	0.221	0.4(0.1,1.4)	0.147	1.4 (0.5,4.3)	0.541	1.1 (0.3,3.5)	0.895	2.7 (0.8, 8.7)	0.103	2.1 (0.6, 7.2)	0.218
Middle	1 (0.3,3.8)	1	0.9(0.2,4.1)	0.927	2.1(0.6,7.2)	0.219	1.3 (0.4,5)	0.68	2.2 (0.7, 7)	0.193	1.4 (0.4, 5.0)	0.549
Higher	1.2(0.3,4.4)	0.774	1.8(0.3,9.1)	0.494	1 (0.3,3.7)	0.94	1.5 (0.4, 6.6)	0.566	3.1 (1, 10.1)	0.054	2.3 (0.6, 9.2)	0.212
Highest	0.8(0.2,3.4)	0.733	1.5 (0.2,12.7)	0.683	2.6(0.5,12.5)	0.246	4.3 (0.4, 44.8)	0.227	1.6 (0.4, 6)	0.497	0.9 (0.1, 6.1)	0.9
**Residence**												
Urban	1		1		1	1	1		1		1	
Rural	1.6 (0.5,4.3)	0.377	3.7(0.9,15.9)	0.079	2.4(0.8,7.5)	0.135	3.2 (0.8,13.5)	0.11	1.1 (0.4, 2.8)	0.866	1 (0.3, 3.6)	0.97
**Literacy level**												
Cannot read at all	1		1		1	1	1		1		1	
Able to read only parts of a sentence	5.5(0.5,60.5)	0.164	8.9(0.6,123.2)	0.108	0.1(0.02,0.9)	0.041	0.1 (0.02,0.9)	0.042	1.3 (0.3, 6.6)	0.729	1.2 (0.2,6.6)	0.819
Able to read whole sentence	1.3(0.5,3.4)	0.528	1.4 (0.5,4.2)	0.541	2.7(1,7)	0.045	3.2 (1.1,8.8)	0.029	1.8 (0.8, 4.2)	0.159	1.6 (0.6, 3.9)	0.356
**Working status**												
For family member/Someone else	1		1		1	1	1		1		1	
Not working	1.1(0.4,3.3)	0.865	0.6(0.2,1.8)	0.411	0.9(0.4,2.2)	0.83	1.05 (0.4,2.7)	0.921	2 (0.8, 5)	0.122	1.6 (0.6, 4)	0.322
Self-employed	0.8(0.3,1.9)	0.602	1.2(0.3,4)	0.799	0.3 (0.1,0.8)	0.02	0.3 (0.1, 0.9)	0.038	1 (0.3,2.9)	0.989	0.8 (0.3, 2.5)	0.737
**Assisted by a nurse**												
No	1		1		1	1	1		1		1	
Yes	1 (0.4,2.4)	0.997	0.9 (0.3,2.7)	0.856	1.2 (0.5,3)	0.628	1.4 (0.5, 3.6)	0.53	2.3 (1.1, 5)	0.038	2.1 (0.9, 5)	0.078
**Frequency of listening to radio**												
Not at all	1		1		1	1	1		1		1	
Less than a week	2(0.7,5.7)	0.21	2(0.5,7.3)	0.306	1.1(0.4,3)	0.907	1 (0.3, 3.1)	0.947	1 (0.4,2.7)	0.957	0.8 (0.3,2.5)	0.746
At least once a week	1.5(0.5,4)	0.448	1.3(0.4,4.7)	0.644	0.8 (0.3,2.2)	0.688	0.5 (0.2, 1.5)	0.228	1.3 (0.5,3.5)	0.612	0.9 (0.3,2.7)	0.8
**Frequency of watching TV**												
Not at all	1		1		1	1	1		1		1	
Less than a week	2.4 (0.8,7.2)	0.123	2(0.5,7)	0.299	0.6 (0.2,1.7)	0.386	0.5 (0.2, 1.6)	0.246	1 (0.4, 2.4)	0.921	0.9 (0.3,2.6)	0.91
At least once a week	1.1(0.3,3.8)	0.853	0.9(0.2,4.4)	0.943	1.2 (0.4,4)	0.733	0.6 (0.1, 3.2)	0.545	1 (0.3,2.7)	0.927	1.3 (0.3,5.7)	0.767
**Random factors**												
Region			40% (19%,65%)				17% (5–41%)				4.13E-33	
EA nested in a region				66% (41%,85%)			63% (41–81%)				40% (16%, 70%)	

Model adjusted for sex, mother’s age, wealth index, mother’s residence, literacy level, whether assisted by a nurse, frequency of listening to radio and watching TV

During the 0–1 month stage, the examination revealed no statistically significant association between the fixed covariates and the prevalence of EBF. However, at 2–3 months, literacy level is associated with EBF. In comparison to mothers who could not read at all, those capable of reading only parts of a sentence exhibited decreased odds of practicing EBF (AOR 0.1, 95% CI 0.002-0.9, P value 0.0420). Conversely, mothers who could read an entire sentence displayed higher odds of adhering to EBF compared to mothers who could not read at all (AOR 3.2, 95% CI 1.1–8.8, P value 0.029). While not attaining statistical significance, mothers not working had almost similar odds of practising EBF compared with mothers working for family member/someone else (AOR 1.05, 95% CI: 0.4-2.7, P value = 0.947). Mothers who were self-employed had lower odds of practising EBF compared to those who worked for family member/someone else (AOR 0.3, 95% CI: 0.1-0.9, P value=0.038). At 4–5 months, none of the fixed covariates exhibited any statistically significant association with EBF practices (Table 2).

The regional and EA variabilities exhibit a decreasing trend as an infant advances in age. Notably, the variability at the EA level surpasses that at the regional level across all age groups. At 0–1 months, regional variability accounts for 40% (95% CI 19–65%) of the observed variation, which then decreases to 17% (95% CI 5–41%) at 2–3 months old. By the time infants reach 4–5 months of age, the regional-level variability becomes negligible. In contrast, at the EA level, a different pattern emerges. For the entire 0–5 months age group, over a quarter of the variability in EBF practices is attributable to EA characteristics, accounting for 29% (95% CI: 19–43%) (data not shown). At 0–1, 2–3, and 4–5 months, there is 66% (95% CI 41–85%), 63% (95% CI 41–81%), and 40% (95% CI 16–70%) of the variability contributed by EA, respectively (Table 2).

## Discussion

This study employed a generalized linear mixed model to assess the variability in EBF practices at the regional level among different age groups of infants. It aimed to identify the determinants of EBF practices within these age bands and examine the extent of variability in EBF practices at both regional and cluster levels.

The findings highlight considerable heterogeneity in EBF practices across the country, particularly with regions along the coastal area of Tanzania mainland exhibiting poorer performance. Notably, the study revealed that maternal factors influence EBF practices at 2–3 months but do not exhibit a significant impact at 0–1 and 4–5 months. Additionally, the analysis demonstrated that a substantial degree of variability in EBF practices can be attributed to regional factors, with an even greater influence stemming from attributes at the cluster level.

In 2012, the World Health Assembly Resolution embraced a comprehensive plan for maternal, infant, and young child nutrition [25]. Among its objectives, this resolution aimed to raise the proportion of EBF during the first 6 months to a minimum of 50%, or for countries already near this threshold, to achieve an annual increase of at least 1.2% by 2025 [25]. Notably, at the national level, Tanzania has seen a consistent upward trend in the proportion of EBF, with proportions climbing from 26% to 1991/2, 32% in 1999, 41% in 2004/5, 50% in 2010, and reaching 59% in 2016 [7]. These figures are significant for two reasons. Firstly, they indicate that Tanzania had already met the Global Nutrition Target (GNT) by 2010, and secondly, the annual increase in percentage points aligns with the GNT recommendations. Therefore, by maintaining its efforts to protect, promote and support EBF on a national scale, Tanzania could feasibly achieve the GNT objective by 2025. However, this positive trajectory is not mirrored when examining EBF practices at the regional level.

Regions along the coastal area appear to be trailing in terms of EBF practices, with prevalence ranging from as low as 10% in Pwani at 0–1 months to 41% in Dar es Salaam. This regional disparity in the proportion of EBF has been a recurrent theme in the literature [8–11, 19, 26]. Several factors contribute to these disparities, including variations in local cultural practices [9], such as the types of food or beverages introduced to infants and the timing of these introductions [27]. Additionally, differences in regional living conditions [10, 19] and the presence of government and non-governmental health organizations interventions [19] have been cited as influencers. To ensure that Tanzania meets GNT by 2025, it is imperative to direct focused efforts towards regions that are lagging behind.

The influence of the mother’s socio-demographic factors on EBF practices was limited when considering smaller infants’ age bands. The only factor that appeared to have any impact was the mother’s literacy level. Conversely, the study revealed a more significant contribution to EBF variability from cluster and regional attributes beyond socio-demographic characteristics. EBF practices may be influenced by cultural practices [9, 20, 27–30], beliefs and practices such as milk expression [9, 13] and abstaining from sexual contact during breastfeeding [16], main economic activity such as farming and breastfeeding mother’s overall workload [9, 11, 14, 20, 29, 31–33], a mother’s alcohol consumption [34], the presence of improved health facilities like those with baby-friendly hospital initiatives [13], counselling on EBF and its quality [9, 31, 32, 34, 35] and environmental factors such as rain availability [14]. These factors may be specific to particular groups of people within smaller geographical areas, such as enumeration areas. In the Tanzanian context, these non-socio-demographic factors may play a more substantial role in influencing EBF practices. A study conducted at a tertiary hospital in Tanzania, focusing solely on HIV-positive women, found no association between EBF and participants’ socio-demographic characteristics [32]. The authors concluded that exposure to counseling could mitigate the impact of socio-demographic characteristics [32]. There is a need to understand the drivers and barriers of EBF practices beyond socio-demographic factors in Tanzania.

The decline in the proportion of EBF as infants age was observed consistently across all regions, even those that exhibited strong performance in EBF. The decline can be attributed to several factors: maternal need to return to work [20, 31, 32], the perception of insufficient milk supply to satiate the baby which is associated with infants appearing fussy or colicky [9, 13, 20, 32, 35]. Peer and societal pressure can exert a significant influence on the premature introduction of solids to an infant’s diet, occurring earlier than the recommended age of 6 months [9, 20]. Interventions are to be developed to address the barriers to EBF as an infant age.

Interventions aimed at addressing the specific barriers that contribute to non-adherence to EBF have demonstrated their effectiveness in increasing EBF prevalence [29, 31]. To further advance our efforts in promoting EBF, it is crucial to conduct targeted studies that delve into the unique barriers and enablers influencing EBF practice within smaller geographic areas and across different infant age groups. These studies can serve as the foundation for the development of tailored interventions designed to identify barriers and recognize enablers. Rigorous research can help us pinpoint the precise obstacles that hinder EBF within specific local context and for infants at various stages of development. Simultaneously, such studies allow us to uncover the factors and conditions that facilitate successful EBF practices, shedding light on what motivates mothers to exclusively breastfeed.

###  Weakness and strength

The study defines the proportion of EBF using the 24-hour recall method, a methodology recommended and endorsed by the WHO [1]. However, it’s worth noting that this method has faced scrutiny due to concerns that it may lead to the reporting of higher EBF proportions [36].

Given the cross-sectional nature of this study, it’s essential to emphasize that causal relationships cannot be inferred. Additionally, the use of secondary data introduced limitations, particularly in terms of access to critical confounding variables like HIV status. Models that account for missing or incomplete data can be considered. For a more comprehensive understanding of determinants across different age bands, a cohort study, involving the longitudinal tracking of mother-infant pairs and the assessment of determinants at various time points, would be a more suitable approach.

Within the constraints of the available data, this study successfully identified determinants within different age groups. However, caution must be exercised when interpreting the findings. The declining proportion of EBF as infants age may have obscured the impact of determinants due to reduced sample sizes at later stages.

Despite these limitations, the study possesses notable strengths. It leveraged nationally representative data, encompassing a substantial portion of clusters from the original dataset, enhancing the robustness of its findings. The utilization of advanced statistical techniques, specifically the generalized linear mixed model, contributes to more accurate estimates of EBF proportions across the entire country. Furthermore, the study’s ability to delineate regional disparities in EBF underscores its potential to inform targeted interventions.

## Conclusion

Infants in Tanzania deserve and should receive the highest standard of care, which unequivocally includes exclusive breastfeeding. While Tanzania exhibits positive progress toward achieving Global Nutrition Target (GNT) objectives, it’s imperative to acknowledge that there are regional disparities and certain regions require targeted intervention. Moreover, the nation remains distanced from attaining the ambitious goal of ensuring that at least 90% of its infants are exclusively breastfed [5]. Moreover, the proportion of infants exclusively breastfed decreases as they grow older across regions, with only a few exceptions. This shortcoming is unacceptable, especially when neighboring countries like Rwanda have achieved some of the world’s highest proportions of exclusive breastfeeding (87%) [37]. Among smaller age groups, socio-demographic attributes have minimal impact on EBF practice. The variability in EBF is primarily influenced by factors at smaller geographical levels. This underscores a resounding call to action for stakeholders at the national, regional, and district levels. There’s an urgent need for concerted efforts to enhance the prevalence of exclusive breastfeeding throughout the country. Furthermore, conducting in-depth studies to uncover the underlying causes of regional disparities, as well as the barriers and facilitators of exclusive breastfeeding within smaller geographic regions and across various infant age groups, should serve as the foundation for well-informed interventions.

### Electronic supplementary material

Below is the link to the electronic supplementary material.

Supplementary Material 1

Supplementary Material 2

## Data Availability

Data used in this study may be accessed at https://dhsprogram.com/ upon permission from DHS Program/ICF international.

## List of Abbreviations

EBF: Exclusive breastfeeding
AIC: Akaike information criterion
AOR: Adjusted odds ratio
CI: Confidence interval
DHS: Demographic and Health Survey
EA: Enumeration area
GLMM: Generalized linear mixed model
GNT: Global nutrition target
TV: Television
WHO: World health organization
