# Supplementary material for: Mapping regional variability of exclusive breastfeeding and its determinants at different infant’s age in Tanzania
Source: BMC Pregnancy Childbirth. 2023 Nov 3;23:769. doi: 10.1186/s12884-023-06076-5 (PMC10623860; doi:10.1186/s12884-023-06076-5)
Supplement: Supplementary file 1 — Supplementary Material 1 [file 12884_2023_6076_MOESM1_ESM.docx]

A crude map was developed to get an overview of the distribution of the EA across the country. The crude map showing the EA distribution of the location of sampled infants aged 0-5 months across the country as collected during the survey was developed and provided as a supplementary material.

**
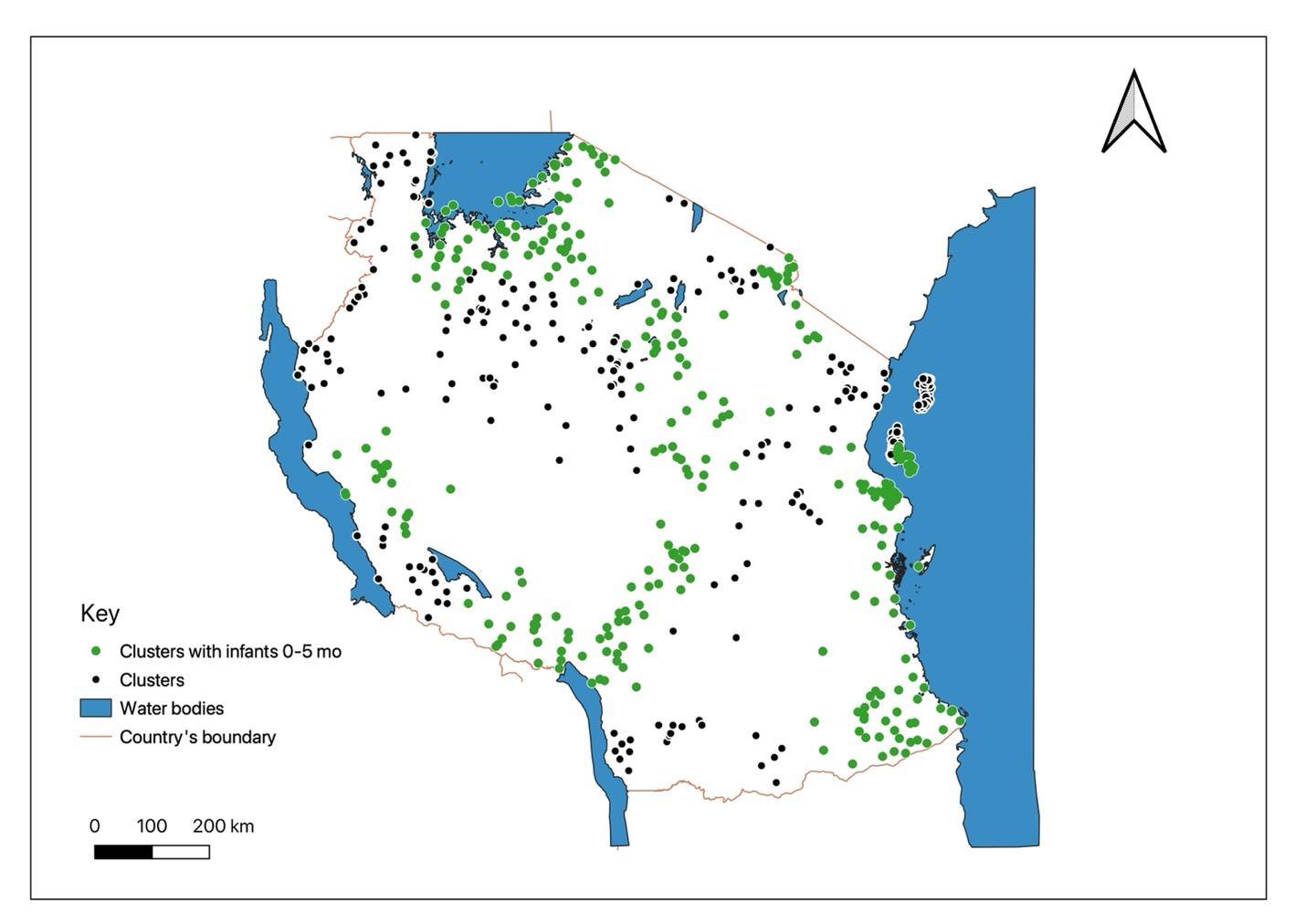
**

**Supplementary Figure 1**: Distribution of EA location for sampled infants aged 0-5 months during the 2015/16 Tanzania Demographic and Health survey
