# Supplementary material for: Mapping regional variability of exclusive breastfeeding and its determinants at different infant’s age in Tanzania
Source: BMC Pregnancy Childbirth. 2023 Nov 3;23:769. doi: 10.1186/s12884-023-06076-5 (PMC10623860; doi:10.1186/s12884-023-06076-5)
Supplement: Supplementary file 2 — Supplementary Material 2 [file 12884_2023_6076_MOESM2_ESM.docx]

Regional fluctuations of EBF over time were presented in a table and provided as a supplementary material.

**Supplementary Table 1*:*** Predicted prevalence of exclusive breastfeeding in Tanzania at the regional level at 0 to 1, 2 to 3, and 4 to 5

|  | **Regional trend** | | |
| --- | --- | --- | --- |
| **Region** | **0-1** | **2-3** | **4-5** |
| Dodoma | **70** | **80** | **47** |
| Arusha | **80** | **77** | **38** |
| Kilimanjaro | **76** | **70** | **38** |
| Tanga | **28** | **24** | **19** |
| Morogoro | **49** | **50** | **35** |
| Pwani | **10** | **9** | **10** |
| Dar es salaam | **41** | **38** | **32** |
| Lindi | **12** | **17** | **16** |
| Mtwara | **52** | **38** | **29** |
| Ruvuma | **31** | **26** | **25** |
| Iringa | **79** | **79** | **31** |
| Mbeya | **94** | **94** | **64** |
| Singida | **80** | **79** | **46** |
| Tabora | **86** | **85** | **68** |
| Rukwa | **69** | **67** | **39** |
| Kigoma | **88** | **86** | **54** |
| Shinyanga | **86** | **85** | **52** |
| Kagera | **89** | **88** | **62** |
| Mwanza | **54** | **57** | **45** |
| Mara | **52** | **51** | **29** |
| Manyara | **68** | **66** | **28** |
| Njombe | **86** | **87** | **54** |
| Katavi | **75** | **65** | **29** |
| Simiyu | **56** | **52** | **30** |
| Geita | **70** | **67** | **40** |
| Kaskazini Unguja | **47** | **38** | **23** |
| Kusini Unguja | **76** | **56** | **28** |
| Mjini Magharibi | **24** | **29** | **19** |
| Kaskazini Pemba | **33** | **20** | **15** |
| Kusini Pemba | **40** | **29** | **16** |
